# Risk for lung cancer in workers exposed to benzidine and/or beta-naphthylamine: a protocol for systematic review and meta-analysis

**DOI:** 10.1186/2046-4053-3-112

**Published:** 2014-10-03

**Authors:** Kimiko Tomioka, Keigo Saeki, Kenji Obayashi, Yuu Tanaka, Norio Kurumatani

**Affiliations:** 1 Department of Community Health and Epidemiology, Nara Medical University, Shijo-cho 840, Kashihara City, Nara 634-8521, Japan; 2 Department of Anesthesiology, Nara Medical University, Shijo-cho 840, Kashihara City, Nara, Japan

**Keywords:** Benzidine, Beta-naphthylamine, Lung cancer, Mortality, Incidence, Occupational exposure, Systematic review, Meta-analysis, Protocol

## Abstract

**Background:**

Risk for lung cancer in workers exposed to benzidine (BZ) and/or beta-naphthylamine (BNA), which are well-known bladder carcinogens, has been examined in many epidemiological studies, but individual epidemiological studies generally lack the power to examine the association between BZ/BNA exposure and lung cancer. We conduct a systematic review and meta-analysis to determine the risk for lung cancer among workers exposed to BZ/BNA occupationally.

**Methods/design:**

Studies will be identified by a MEDLINE, EMBASE, CDSR, and CINAHL search and by the reference lists of articles/relevant reviews. Eligible studies will be cohort and case-control studies that report occupational BZ/BNA exposure and the outcome of interest (lung cancer death/incidence). The method of meta-analysis will be used to combine standardized mortality ratios (SMRs) and/or standardized incidence ratios (SIRs) from retrospective and prospective cohort studies and odds ratios (ORs) from case-control studies. Two reviewers will independently screen articles, extract data, and assess scientific quality using standardized forms and published quality assessment tools tailored for each study design. Overall pooled risk estimates and their corresponding 95% confidence intervals (CIs) will be obtained using random effects model. This systematic review and meta-analysis will be conducted following the Meta-analysis of Observational Studies in Epidemiology (MOOSE) guidelines, and results will be reported according to the Preferred Reporting Items for Systematic Reviews and Meta-Analyses (PRISMA) statement.

**Discussion:**

This review will identify and synthesize studies of the association between occupational BZ/BNA exposure and lung cancer. The findings will help to identify whether BZ/BNA could cause lung cancer and might indicate whether workers with exposure to BZ/BNA have a need for preventive measures against non-urological cancer besides bladder cancer.

**Systematic review registration:**

PROSPERO CRD42014010250

## Background

Benzidine (BZ) and beta-naphthylamine (BNA), which belong to aromatic amines, are classified by the International Agency for Research on Cancer (IARC) as definite human carcinogens (Group 1) on the basis of sufficient evidence of bladder cancer in animals and human beings [1,2]. In addition to the carcinogenicity on the bladder, some occupational epidemiological studies have observed the risk for cancer at sites other than the bladder (i.e., lungs [3-5], esophageal [4], liver, gallbladder, and bile duct [6], intestines and larynx [7], and lymphohematopoietic [8]). However, epidemiological evidence for BZ- and/or BNA-induced cancer in sites other than the bladder is not as strong as for bladder cancer itself. Reasons for this include the small number of cases among the populations studied, which diminishes the power of the statistical analyses, and inconsistency between studies regarding tumor sites.

BZ and BNA were tested for carcinogenicity by many routes (e.g., oral, subcutaneous injection, intraperitoneal injection, and inhalation) in many animal species (e.g., mice, rats, hamsters, rabbits, and dogs), and a number of animal studies indicate that exposure to these chemicals can increase the incidence of a variety of tumors [1,2,9]. With regard to lung cancers, some studies have reported that oral administration of BZ produced a high incidence of lung tumors in mice [10], BNA significantly increased the incidence of lung adenomas following its intraperitoneal injection to mice [11], and BNA caused lung tumor multiplicity following its administration by gavage to mice [12]. In regard to cancer-causing mechanisms in humans, it is thought that aromatic amines undergo metabolic activation by *N*-hydroxylation of the exocyclic amine group to form the proposed arylnitrenium ion, which is the critical metabolite implicated in toxicity and DNA damage [13,14]. The arylhydroxylamine metabolite can form a DNA adduct. Because DNA adducts derived from the genotoxic metabolites are recognized as representative early biomarkers of cancer risk [15,16], the identification and measurement of chemical-specific DNA adducts in the target tissue are the most relevant findings for risk assessment [16,17]. In the case of 4-aminobiphenyl, which is a prototypical aromatic amine and is present in significant quantities in tobacco smoke, DNA adducts were first detected in human urinary bladder tissue biopsy samples and exfoliated urothelial cells [18,19]. Subsequently, adducts were detected in human lung tissues obtained by surgery or autopsy [20]. The experimental evidence of lung cancer for BZ and BNA in mice [10-12] and the detection of DNA adducts of an aromatic amine from human lung tissues [20] suggest that BZ and BNA have the potential to cause lung cancer in humans.

For occupational epidemiological studies, several studies have reported significantly increased risk of lung cancer [3-5]. A cohort study of 1,287 American BNA production workers [3] found a statistically significant excess risk of death due to lung cancer. The lung cancer risk was elevated in those with the longest working experience, but the authors could not investigate exposure-response trends because more than 80% of the study members were short-term (less than 1 year) workers. A cohort study of 4,581 Russian aniline-dye production workers [4] showed a significantly increased risk for lung cancer incidence among workers employed in BZ- or BNA-exposed jobs. But lung cancer risk was also significantly elevated among workers exposed only to other chemicals and among maintenance workers. A more recent cohort study of 374 US workers exposed to BNA [5] also observed a statistically significant excess risk of death due to lung cancer. But the risk for lung cancer was statistically significant not only in the highest BNA exposure risk group, but also among subjects hired after 1972, employed less than 1 year, or with the lowest BNA exposure risk. The statistical power of those individual studies was thus inadequate to allow a proper interpretation of the effect of BZ and/or BNA on the risk of lung cancer. Additionally, to our knowledge, there have been no meta-analyses of lung cancer risk in workers exposed to BZ and/or BNA.

To examine whether occupational exposure to BZ and/or BNA is associated with risk of lung cancer, we conduct a systematic review and meta-analysis with data from occupational epidemiological studies regarding the association of BZ and/or BNA with lung cancer risk.

## Methods/design

This systematic review and meta-analysis will be conducted following the Meta-analysis of Observational Studies in Epidemiology (MOOSE) guidelines [21] and will be reported in accordance with the Preferred Reporting Items for Systematic Reviews and Meta-Analyses (PRISMA) statement [22]. This systematic review protocol was registered with the International Prospective Register of Systematic Reviews (PROSPERO) database (registration number: CRD42014010250).

### Inclusion and exclusion criteria

PRISMA [22] asks authors to describe eligibility criteria using the PICOS reporting system (which describes the participants, interventions, comparisons, outcome(s), and study design of the systematic review). This review will include observational studies, so we will not require using an intervention for inclusion. Therefore, in accordance with the MOOSE guidelines [21], we will describe eligibility criteria by means of the participants/population, exposure(s), comparator(s)/control, outcome(s), and study design (PECOS).

### Participants/population

We will include subjects with unequivocal evidence of occupational exposure to BZ and/or BNA such as dyestuff workers, workers of BZ/BNA manufacturing plant, and rubber industry workers exposed to BZ/BNA, which was present as a contaminant in antioxidants used in manufacturing. Subjects who worked at the same factory and who were exposed to neither BZ nor BNA will be excluded.

### Exposure

For cohort studies, ascertainment of exposure to BZ and/or BNA should be based on written records of exposure measurements or work history. For case-control studies, BZ/BNA exposure should be ascertained by secure record (i.e., surgical records), structured interview where blind to case/control status, interview not blinded to case/control status, or written self-report.

### Comparator(s)/control

For cohort studies, use of a comparator is not a requirement for inclusion. For case-control studies, control group will include subjects who have no history of lung cancer.

### Outcome

Our outcome will be lung cancer death and/or lung cancer incidence based on clinically confirmed diagnosis (i.e., death certificates, cancer registry or other national recording system, or hospital or doctors’ records). Effect measure will include the standardized mortality ratio (SMR), standardized incidence ratio (SIR), and odds ratio (OR) for the association between BZ/BNA exposure and lung cancer risk. The SMR and SIR will be based on an external comparison group (i.e., national or regional population), and the OR based on a population- or hospital-based control group.

### Study design

Eligible studies will be comparative observational studies that report occupational BZ/BNA exposure and the outcome of interest (lung cancer death and/or lung cancer incidence). Retrospective cohort studies (also known as historical cohort studies), prospective cohort studies, and case-control studies will be included in this review.

### Search strategy

The search strategies will be carried out by the research team and an expert librarian. No language restriction will be enforced conditional to the provision of an English abstract. A date restriction will not be imposed. We will search the following electronic databases, from inception, using the same search strategy with alterations as appropriate for each database: MEDLINE, Excerpta Medica DataBase (EMBASE), Cochrane Database of Systematic Reviews (CDSR), and Cumulative Index to Nursing and Allied Health Literature (CINAHL). Search terms will include controlled vocabulary and text-words, and details of the search strategy for MEDLINE are provided in an additional file (see Additional file 1). Additional studies will be identified from the reference list of articles and relevant reviews. We will attempt to identify unpublished studies by contacting authors of included studies.

### Study selection

Study selection will be done in two stages. First, all titles and abstracts will be independently screened by two reviewers against the inclusion/exclusion criteria to identify potentially relevant studies. Studies that do not meet specific inclusion/exclusion criteria will be rejected at this stage, and the reason for rejection will be recorded. Second, the full text articles of all remaining studies will be obtained and independently assessed for inclusion by two reviewers. Disagreements between the two reviewers will be resolved by discussion, with the involvement of a third reviewer where agreement cannot be reached. Multiple reports of the same study will be counted only once; the record containing the greatest amount of information (for example, largest sample size or longest follow-up period) will be retained. A flow chart showing details of studies included and excluded at each stage of the study selection process will be produced following the PRISMA template.

### Data extraction

Data from all included studies will be extracted independently by two reviewers into a standardized data collection form (see Additional file 2) which will be piloted on a sample of five studies and then modified if necessary before full data extraction begins. Discrepancies will be resolved by discussion, with the involvement of a third reviewer where necessary. For cohort studies, we will extract the number of observed deaths or cases, the number of expected deaths or cases, the effect measure (i.e., SMR or SIR) and the 95% confidence interval (CI) for lung cancer (essential), all cancers (if available), and bladder cancer (if available). We want to collect data on all cancers and bladder cancer as potential sources of heterogeneity between the included studies. For the studies for which the 95% CI is not reported, we will calculate them by the exact probabilities of the Poisson distribution using the observed deaths/cases and expected deaths/cases reported in the articles [23]. If a study uses both national and regional populations to compute the expected deaths/cases, results based on the regional statistics will be used because a regional population of study subjects is preferable to the national population for controlling geographic differences in disease incidence [24]. For case-control studies, we will extract the number of the case group and control group, the OR, and the 95% CI for lung cancer from each included study. For all included studies, data on the following study characteristics will be also extracted where available: year of publication, proportion of male, mean age, duration of follow-up, duration of exposure, country and geographic area, industry type, occupational exposure to chemicals including BZ and BNA, information of cigarette smoking, study sponsorship, and information relating to quality assessment. In addition, data on national incidence rates for lung cancer will be obtained from GLOBOCAN 2012 estimates [25] and data on national prevalence of cigarette smoking from WHO Report on the Global Tobacco Epidemic 2013.

### Quality assessment

Quality will be assessed by two reviewers and discrepancies resolved by discussion.

For case-control studies, we will use the Newcastle-Ottawa scale (NOS) [26]. This assessment scale consists of eight items that are categorized into three major components: selection, comparability, and exposure. For cohort studies, a modified version of the NOS [27] will be used. This modified NOS was developed for assessing the quality of occupational cohort studies and includes five quality components: representativeness of the exposed cohorts, exposure assessment/reporting, comparability of exposed and non-exposed cohort, assessment of outcome, and adequacy of follow-up.

### Data synthesis

We will perform meta-analysis to obtain the weighted average (pooled) of the SMR or SIR for cohort studies and the OR for case-control studies by using the Comprehensive Meta-Analysis (version 2.0) software. Overall pooled estimates of the SMR, SIR, and OR, denoted “meta-SMR”, “meta-SIR”, and “meta-OR”, respectively, together with associated 95% CIs, will be calculated based on random effects model [28]. Heterogeneity among included studies will be investigated using the *I*^2^ statistic (where thresholds of <25% will be taken to suggest low heterogeneity, <50% to suggest moderate heterogeneity, and >75% to suggest high heterogeneity [29]) and Cochran’s *Q* test (where the significance level for chi squared will be set at *P* = 0.1). Publication bias will be investigated by visual inspection of Begg’s funnel plots and formally tested using Egger’s regression asymmetry method [30].

We will use subgroup analyses to attempt to explain any observed between-study heterogeneity. The covariates considered will be geographic region, cohort/sample size, follow-up period, year of starting the production of BZ/BNA, type of exposure to BZ/BNA, type of industry, occupational exposure to lung carcinogen, SMR/SIR for all cancers and bladder cancer, national prevalence of cigarette smoking, and national incidence rate for lung cancer.

For sensitivity analyses, we will analyze key outcomes separately by categories of the assessed study quality variables to ascertain whether there are any relations with quality and outcome. Additionally, we will assess the influence of individual studies on the overall meta-SMR (meta-SIR or meta-OR) by re-estimating the overall effect after omitting each study in turn. We will also conduct cumulative meta-analysis in the order of publication year to find the starting point of risk estimate becoming statistically significant and clarify the variation tendency [31].

## Discussion

This systematic review will identify and synthesize evidence of the association between exposure to BZ/BNA and lung cancer in humans. The findings of this review will help to identify whether carcinogenic aromatic amines such as BZ and BNA could cause lung cancer and provide industrial physicians and policy makers with information on whether workers with exposure to these carcinogenic chemicals have a need for health measures aimed at preventing non-urological cancer, especially lung cancer.

We are aware that randomized controlled trials (RCTs) are considered to provide the strongest evidence regarding an intervention [32,33] and that observational studies are subject to high risk of bias due to potential outcome confounding [21]. However, it is impossible to conduct RCTs about how a human carcinogen chemical causes additional carcinogenicity because exposing subjects to harmful chemical is unethical. Therefore, only data from observational studies are available for etiological study about well-known human carcinogen. These limitations will be discussed in length in our review giving special attention to the possible bias and estimates’ precision of studies conducted in non-randomized controlled designs. For another major concern in interpreting our findings, we think that smoking and occupational exposure to chemicals other than BZ and BNA are important confounders for our study. It is especially important to confirm whether subjects have occupational exposure to chemicals known to be carcinogenic for the lung. Therefore, we will gather as much information as possible about smoking and occupational exposure to chemicals other than BZ and BNA and conduct subgroup analysis by these important confounders.

With this systematic review, we hope to provide a better understanding of the long-term health effects for workers exposed to carcinogenic chemicals.

## Abbreviations

BZ: benzidine; BNA: beta-naphthylamine; CDSR: Cochrane Database of Systematic Reviews; 95% CI: 95% confidence interval; CINAHL: Cumulative Index to Nursing and Allied Health Literature; EMBASE: Excerpta Medica DataBase; IARC: International Agency for Research on Cancer; MOOSE: Meta-analysis of Observational Studies in Epidemiology; NOS: Newcastle-Ottawa scale; OR: odds ratio; PRISMA: Preferred Reporting Items for Systematic Reviews and Meta-analyses; PROSPERO: International prospective register of systematic reviews; RCT: randomized controlled trial; SIR: standardized incidence ratio; SMR: standardized mortality ratio.

## Competing interests

The authors declare that they have no competing interests.

## Authors’ contributions

KT, KS, KO, YT, and NK participated in the design of the protocol and helped to draft the manuscript. All authors read and approved the final manuscript.

## Authors’ information

KT, KS, and KO are Associate Professors of Community Health and Epidemiology at Nara Medical University School of Medicine.

YT is an Associate Professor of Anesthesiology at Nara Medical University School of Medicine, a member of Cochrane Anaesthesia Group, and an author who has a review published in The Cochrane Library.

NK is Professor of Community Health and Epidemiology at Nara Medical University School of Medicine.

## Supplementary Material

Additional file 1 **Search strategy.** Details of the search strategy for MEDLINE. Click here for file

Additional file 2 **Data collection form.** Data from all included studies will be extracted independently into this standardized data collection form. Click here for file
