# Supplementary material for: Risk for lung cancer in workers exposed to benzidine and/or beta-naphthylamine: a protocol for systematic review and meta-analysis
Source: Syst Rev. 2014 Oct 3;3:112. doi: 10.1186/2046-4053-3-112 (PMC4186647; doi:10.1186/2046-4053-3-112)
Supplement: Additional file 1 — Search strategy. Details of the search strategy for MEDLINE. [file 2046-4053-3-112-S1.pdf]

MEDLINE Search:

#1	(benzidines OR benzidine) OR (biphenyldiamines OR biphenyldiamine) OR (bianilines OR bianiline) OR 2-naphthylamine OR beta-naphthylamine OR 2-aminonaphthalene OR dyestuff*[tiab]
#2	mortality OR incidence OR morbidity OR "cause of death" OR risk OR "adverse effects" OR epidemiology
#3	neoplasms OR carcinogen* OR cancer OR tumor OR tumour OR carcinoma
#4	work[tw] OR works*[tw] OR worka*[tw] OR worke*[tw] OR workg*[tw] OR worki*[tw] OR workl*[tw] OR workp*[tw]
#5	industr*[tiab] OR manufactur*[tiab] OR occupation*[tiab] OR employ*[tiab] OR product*[tiab] OR factory*[tiab] OR factories*[tiab]
#6	"occupational exposure"[MeSH] OR "occupational diseases"[MeSH]
#7	#4 OR #5 OR #6
#8	#1 AND #2 AND #3 AND #7
#9	"animals"[MeSH Terms:noexp]
#10	#8 NOT #9
