# Supplementary material for: Risk for lung cancer in workers exposed to benzidine and/or beta-naphthylamine: a protocol for systematic review and meta-analysis
Source: Syst Rev. 2014 Oct 3;3:112. doi: 10.1186/2046-4053-3-112 (PMC4186647; doi:10.1186/2046-4053-3-112)
Supplement: Additional file 2 — Data collection form. Data from all included studies will be extracted independently into this standardized data collection form. [file 2046-4053-3-112-S2.pdf]

Data collection form

Notes:

- Be consistent in the order and style used to describe each report.
- Record any missing or unclear information so to indicate that the information was not found in the study report(s) but not forgotten.

General Information

Study ID (surname of first author and year of publication)	(e.g. Tomioka 2013)
Date form completed	(dd/mm/yyyy)
Name of person extracting data	
Reference citation	(e.g.Medline)
Type of publication	(e.g. full report, abstract, letter)
Notes:	

Study eligibility

Study Characteristics	Eligibility criteria	Eligibility criteria met?			Location in text or source (pg & fig/table)
		Yes	No	Unclear/ unspecified	
Type of study	Retrospective cohort study	☐	☐	☐	
	Prospective cohort study	☐	☐	☐	
	Case–control study	☐	☐	☐	
Participants	Employees exposed to BZ and/or BNA in the workplace.	☐	☐	☐	
Types of outcome measures	Lung cancer death and/or lung cancer incidence	☐	☐	☐	
	Clinically confirmed diagnosis	☐	☐	☐	
	Standardized mortality ratio	☐	☐	☐	
	Standardized incidence ratio	☐	☐	☐	
	Odd ratio	☐	☐	☐	
INCLUDE ☐	EXCLUDE ☐	UNCERTAIN ☐			
Reason for exclusion					
Notes:					

DO NOT PROCEED IF STUDY EXCLUDED FROM REVIEW

Characteristics of included studies

Study design/characteristics

	Descriptions as stated in report/paper	Location in text or source (pg & fig/table)
Study aim/objective		
Study subjects		
Reference group	National population ☐<br>Regional population ☐<br>Population-based control group ☐<br>Hospital-based control group ☐<br>Other ( )	
Cancer identification	Cancer registry ☐ Death certificate ☐<br>Medical record ☐ Other ( )	
Country		
Industry type		
Exposure to BZ		
Exposure to BNA		
Exposure assessment/ reporting		
Occupational exposure to other chemicals		
Information of smoking		
Related papers (first author, year)		
Potential confounders adjusted		
Study sponsorship	Yes ☐ No ☐ Unclear ☐	
Notes:		

Study/sample characteristic

	Reported	Not reported	Unclear	Not applicable	If "reported" is checked, the value is recored.
Sample size: recruited					
Sample size: analysed					
Number of total person-years					
Starting year of follow-up					
Ending year of follow-up					
Loss to follow-up (%)					
Proportion males (%)					
Average age					
Starting year of BZ/BNA exposure					
Ending year of BZ/BNA exposure					

Outcomes (Standardized mortality ratio: SMR)

	Reported	Not reported	Unclear	Not applicable	If "reported" is checked, the following columns are completed.				
					No of observed deaths	No of expected deaths	SMR	95% CI (lower limit)	95% CI (upper limit)
**For lung cancer**									
Males									
Females									
All subjects									
**For all cancers**									
Males									
Females									
All subjects									
**For bladder cancer**									
Males									
Females									
All subjects									

Outcomes (Standardized incidence ratio: SIR)

	Reported	Not reported	Unclear	Not applicable	If "reported" is checked, the following columns are completed.				
					No of observed cases	No of expected cases	SIR	95% CI (lower limit)	95% CI (upper limit)
**For lung cancer**									
Males									
Females									
All subjects									
**For all cancers**									
Males									
Females									
All subjects									
**For bladder cancer**									
Males									
Females									
All subjects									

Outcomes (Odd ratio: OR)

OR for lung cancer	Reported	Not reported	Unclear	Not applicable	If "reported" is checked, the following columns are completed.				
					No of cases	No of controls	OR	95% CI (lower limit)	95% CI (upper limit)
**Males**									
Crude OR									
Adjusted OR									
**Females**									
Crude OR									
Adjusted OR									
**All subjects**									
Crude OR									
Adjusted OR									
